# The structures of *E. coli* NfsA bound to the antibiotic nitrofurantoin; to 1,4-benzoquinone and to FMN

**DOI:** 10.1042/BCJ20210160

**Published:** 2021-07-09

**Authors:** Martin A. Day, David Jarrom, Andrew J. Christofferson, Antonio E. Graziano, J. L. Ross Anderson, Peter F. Searle, Eva I. Hyde, Scott A. White

**Affiliations:** 1 School of Biosciences, University of Birmingham, Edgbaston, Birmingham B15 2TT, U.K.; 2 Institute for Cancer and Genomic Sciences, University of Birmingham, Edgbaston, Birmingham B15 2SY, U.K.; 3 School of Science, RMIT University, Melbourne, Victoria 3000, Australia; 4 School of Biochemistry, University of Bristol, Bristol BS8 1TD, U.K.

**Keywords:** antibiotic, flavoprotein, nitroreductase, oxidation–reduction

## Abstract

NfsA is a dimeric flavoprotein that catalyses the reduction in nitroaromatics and quinones by NADPH. This reduction is required for the activity of nitrofuran antibiotics. The crystal structure of free *Escherichia coli* NfsA and several homologues have been determined previously, but there is no structure of the enzyme with ligands. We present here crystal structures of oxidised *E. coli* NfsA in the presence of several ligands, including the antibiotic nitrofurantoin. Nitrofurantoin binds with the furan ring, rather than the nitro group that is reduced, near the N5 of the FMN. Molecular dynamics simulations show that this orientation is only favourable in the oxidised enzyme, while potentiometry suggests that little semiquinone is formed in the free protein. This suggests that the reduction occurs by direct hydride transfer from FMNH^−^ to nitrofurantoin bound in the reverse orientation to that in the crystal structure. We present a model of nitrofurantoin bound to reduced NfsA in a viable hydride transfer orientation. The substrate 1,4-benzoquinone and the product hydroquinone are positioned close to the FMN N5 in the respective crystal structures with NfsA, suitable for reaction, but are mobile within the active site. The structure with a second FMN, bound as a ligand, shows that a mobile loop in the free protein forms a phosphate-binding pocket. NfsA is specific for NADPH and a similar conformational change, forming a phosphate-binding pocket, is likely to also occur with the natural cofactor.

## Introduction

**Figure 1.**
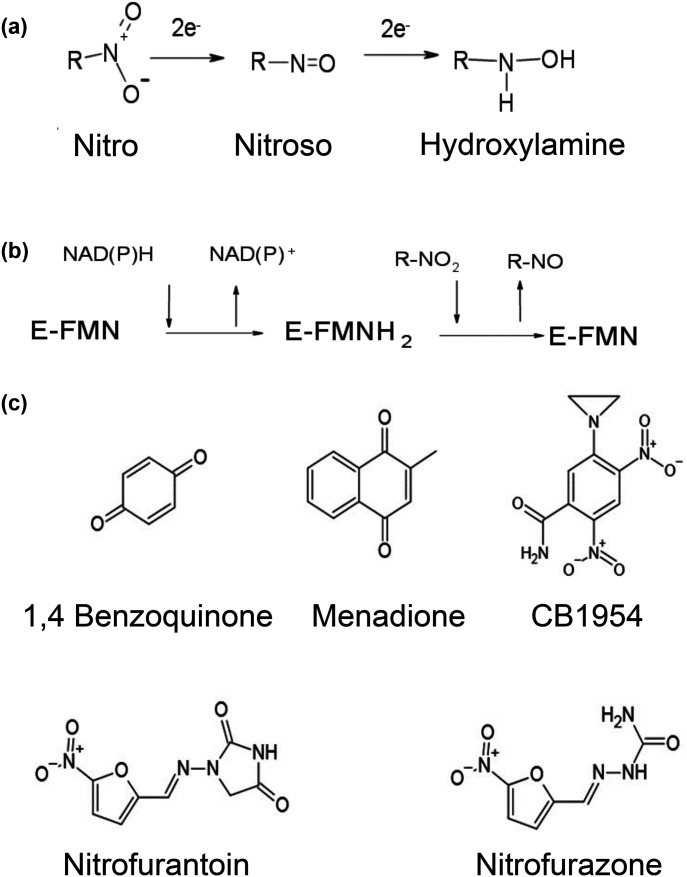
Reaction and substrates of nitroreductases. (**a**) Reduction of nitro groups by two-electron steps to nitroso and then to hydroxylamine derivatives. (**b**) Substituted enzyme (ping-pong) mechanism of nitroreductases. In the first step, the FMN cofactor of the enzyme is reduced to FMNH_2_. In the second step the enzyme reduces the substrate. (**c**) Structures of substrates of NfsA studied. Top: 1,4-Benzoquinone; Menadione, CB1954; Bottom: Nitrofurantoin, nitrofurazone.

Nitrofurans are often used as antibiotics or antimicrobial agents (reviewed in [1]). Nitrofurantoin is used against simple urinary tract infections, while nitrofurazone is used topically against skin infections in patients with burns. With increasing resistance to other antibiotics, interest in the use of nitrofuran antibiotics is growing as, despite over 60 years of use, there is little resistance to these; however, both nitrofurantoin and nitrofurazone are poorly soluble. Their mode of action is unusual as they are reduced by bacterial nitroreductases to nitroso compounds and then to reactive hydroxylamines, that attack a wide range of biological molecules [2,3], Figure 1a. The first step of resistance in *Escherichia coli* to these antibiotics is mutation of NfsA, the major oxygen insensitive nitroreductase, with mutation of NfsB, the minor oxygen insensitive nitroreductase, as a later step [4,5]. Resistance to nitrofurantoin can also occur by up-regulation of efflux pumps in the bacteria [6], or to mutations in *ribE*, lumazine synthase [7], which is essential for the biosynthesis of the FMN cofactor for the enzymes.

NfsA and NfsB belong to two families of nitroreductases that occur in a large number of bacteria (reviewed in [8]). These two families form part of a very large superfamily of proteins, with over 20 000 sequences known to date, that catalyse a diverse range of reactions [9,10]. NfsA and NfsB are FMN-containing proteins that use NAD(P)H to reduce quinones and nitroaromatics, with a substituted enzyme (ping-pong) mechanism [11,12], Figure 1b. They react via two-electron steps, without the formation of free radicals, and hence are classified as oxygen insensitive, as opposed to oxygen sensitive nitroreductases that give one-electron radical intermediates [13]. NfsA uses NADPH preferentially as the cofactor [11,14], whereas NfsB can use either NADH or NADPH with similar affinity [12,15]. Both enzymes are up-regulated under redox stress [16–18] but, apart from FRP, an NfsA analogue, from *Vibrio fisherii* [19] and Frase 1, an NfsB analogue, from *Vibrio harveii* [20], which both reduce FMN to produce light, their true substrates and roles *in vivo* are not known. In addition to their use with antibiotics, the ability of the enzymes, particularly NfsB, to reduce nitroaromatics to cytotoxic hydroxylamines has been used for cancer gene therapy with prodrugs such as CB1954 (reviewed in [21]), in cell ablation studies [22–24] and in bio-remediation of TNT [25,26].

The structure of *E. coli* NfsA has been determined in the absence of substrate by Kobori et al. (1F5V) [27] but there are no reported structures of the protein with substrates or inhibitors. Structures of FRP (1BKJ) [28] and of six other NfsA homologues have also been determined to date (3N2S, 1ZCH, 5UU6, 5HDJ, 5HEI, 3EOF). Only that of FRP has been determined in the presence of a ligand, the inhibitor, NAD^+^, (2BKJ) [29]. This is in a folded, ring-stacked, conformation, with the pyrophosphate group close to the FMN, and the N4 of the nicotinamide ring over 10 Å from the FNM N5 and hence in an inactive conformation. No structure of any enzyme with bound nitrofurantoin has been reported to date. To help in the design of other nitrofuran antibiotics that are more soluble and so able to target bacteria elsewhere in the body, we determined the structure of NfsA from *E. coli* in complex with nitrofurantoin and, separately, with the substrate 1,4-benzoquinone, with hydroquinone and with a second FMN acting as an inhibitor. This work complements our previous studies of the structures of *E. coli* NfsB with nitrofurazone [30], and with nicotinic acid, a mimic of the cofactor headpiece [31], and studies of *Enterobacter cloacae* NR (the NfsB homologue) with NAAD and ligands by Miller et al. [32,33]. The structures of nitrofurazone, nitrofurantoin and the substrates studied are shown in Figure 1c.

## Experimental methods

### Protein expression and purification

*E. coli* NfsA was over expressed in *E. coli* BL21 (λDE3) without any tags, from the pET 24 derivative pPS1341A1, encoding NfsA under the control of a T7 promoter, as described in Vas et al. [14]. It was purified as described previously, using ammonium sulfate precipitation, hydrophobic interaction chromatography on phenyl sepharose, ion-exchange chromatography on Q sepharose, followed by size exclusion chromatography on Sephacryl 200 or Superdex 75.

Protein concentrations were estimated by Bradford assay [34] or by determining the absorbance at 280 nm where both the protein and the cofactor absorb, and correcting for excess FMN by measuring the absorbance at 454 nm, where only FMN absorbs. The molar absorbances used were 12 200 M^−1 ^cm^−1^ for FMN at 454 nm, 20 970 M^−1 ^cm^−1^ for FMN at 280 nm and 31 190 M^−1 ^cm^−1^ for NfsA at 280 nm, based on its amino acid composition [35].

### Steady-state enzyme assays

Steady-state kinetic assays were monitored spectrophotometrically, over 1–2 min, as described previously [30]. Experiments were performed in 10 mM Tris–HCl pH 7.0, at 25°C, unless otherwise stated. Reactions were initiated by the addition of a small quantity of enzyme (∼10 nM). Nitrofurazone, nitrofurantoin and menadione were dissolved in 90% DMSO, 10 mM Tris–HCl pH 7.0; kinetic experiments with these reagents included a final concentration of 4.5% DMSO. Those with nitrofurantoin also included 50 mM NaCl. CB1954 was dissolved in a 2 : 7 mixture of NMP:PEG 300; kinetic experiments with CB1954 included final concentrations of 1.1% NMP and 3.9% PEG 300. For menadione, and CB1954, the reactions were coupled to the reduction in cytochrome c, present at 100 µM, to increase the absorbance change of the reaction and hence to measure rates more accurately at low NADPH or substrate concentration. This was not possible for 1,4-benzoquinone as it reacts with cytochrome c directly.

For nitrofurazone, reactions were measured at either 420 nm or 440 nm, using the molar absorbance change of 4300 and 880 M^−1 ^cm^−1^ for the reaction, respectively. For nitrofurantoin, reactions were measured at 400 nm or 420 nm, with molar absorbance changes of 13 807 or 7970 M^−1 ^cm^−1^. For CB1954, reactions were measured at 420 nm, with molar absorbance change 1200 M^−1 ^cm^−1^, or, if coupled to cytochrome c, measured at 550 nm using 13 500 M^−1 ^cm^−1^ per NADPH. For 1,4-benzoquinone, the reaction was measured at 340 nm, using the molar absorbance of NADPH 6 200 M^−1 ^cm^−1^

For each reaction, the initial rate (*v*_i_) was calculated for a range of concentrations of one substrate (A) whilst keeping the concentration of the other substrate (B) constant. To obtain the full *K*_m_ parameters for each substrate, and the overall *k*_cat_, kinetic measurements were collected over a range of concentrations of both substrates and fitted to eqn 1, describing the overall kinetics for a ping-pong reaction, by nonlinear regression with equal weighting all points, using the programme Sigmaplot (Systat Software, San Jose, CA). [E] is the enzyme concentration.

1 $$\frac{v_{i}}{\left[\text{E}\right]}=\;\frac{k_{\mathrm{cat}}[\text{A}][\text{B}]}{K_{\mathrm{mA}}[\text{B}]+K_{\mathrm{mB}}[\text{A}]+[\text{A}][\text{B}]}$$

For studies of FMN inhibition, the buffer used included 50 mM NaCl, to minimise the effect of increase in ionic strength on the addition of the inhibitors. Initial reaction rates, *v*_i_, were measured for a range of concentrations of one substrate in the presence of a fixed concentration of the other substrate, with and without the inhibitor [I], at two concentrations of inhibitor. All the data for both substrates were fitted simultaneously to eqn 2, describing inhibition of both halves of the ping-pong reaction, with *K*_iA_ and *K*_iB_ being the dissociation constants of the inhibitor in each half reaction. The data were also fitted for inhibition of only one half of the reaction; i.e. competition with only substrate A or only substrate B and the fits compared.

2 $$\frac{v_{i}}{\left[\text{E}\right]}=\;\frac{k_{\mathrm{cat}}[\text{A}][\text{B}]}{K_{\mathrm{mA}}[\text{B}]\left(1+\;\frac{\left[I\right]}{K_{\mathrm{iA}}}\right)+\;K_{\mathrm{mB}}[\text{A}]\left(1+\frac{\left[I\right]}{K_{\mathrm{iB}}\;}\right)+\;[\text{A}][\text{B}]}$$

### X-ray crystallography

All crystals were grown by a sitting-drop method. Purified NfsA was concentrated to between 10 and 16 mg/ml, and then dialysed into 100 mM imidazole, pH 7.0. The mother liquor for NfsA crystallization contained 50–200 mM imidazole pH 7 as a buffer and 18–34% PEG 3 000 (Fluka Analytical, St. Gallen, Switzerland) as a precipitant, in the presence of appropriate ligands. To obtain crystals of complexes, the following concentrations of ligand were added to the mother solution: nitrofurantoin, 3.5 mM, hydroquinone 25 mM, 1,4-benzoquinone 10 mM, Crystals appeared within 24 h and generally reached full size within 48 h. To cryo-protect the crystals, they were soaked in mother liquor containing increasing concentrations of either ethylene glycol or DMSO, lowering the concentration of the PEG precipitant alongside each incremental increase in cryo-protectant. The crystals were then flash-cooled in liquid nitrogen.

Data were collected either at the European Synchrotron Radiation facility, in Grenoble, or on a Rigaku 007HF generator with a Saturn CCD detector mounted on a 4-circle kappa goniometer.

Diffraction images were indexed, integrated and processed using MOSFLM [36], iMOSFLM [37] or XDS [38]. Datasets were combined and scaled using POINTLESS and SCALA [39] and data quality was assessed using XTRIAGE [40]. All structures were solved by molecular replacement with PHASER [41], using the published NfsA structure pdb entry 1F5V for NfsA [27] as the starting model. Structures were refined using REFMAC5 [42] and PHENIX [40]. Models were built and modified using Coot [43]. Some models were further refined using the PDB-REDO automatic server [44]. Final models were validated using MOLPROBITY [45] and POLYGON [46]. The structural figures were drawn using UCSF Chimera 1.13.1 [47].

### Redox titrations

Potentiometric titrations were performed using a modified quartz EPR OTTLE cell equipped with platinum working and counter electrodes and an Ag/AgCl reference electrode (BASi, U.S.A.) in concert with a Biologic SP-150 potentiostat (Bio-logic, France) and Cary 60 UV/visible spectrometer (Agilent, U.S.A.), monitoring the absorbance of the solution. Titrations were performed with 80 μM NfsA in 50 mM phosphate buffer, pH 7.5, 500 mM KCl, 10% glycerol, in the absence of redox mediators as the protein was found to react with these. The potential was initially changed from −50 mV to −350 mV in steps of 20 mV and full UV spectra taken after equilibration at each step, to observe the reduction in the protein. The titration was then reversed, changing the potential to −335 mV and then from −320 mV to −90 mV, in steps of 20 mV and finally to −60 mV. The reduction potentials were determined by fitting the absorbance at 458 nm as a function of potential either to two single one-electron Nernst equations (equation 3) or to a concerted two-electron Nernst equation (equation 4) using Sigmaplot. First, each half titration was fitted separately, to determine the absorbance of the oxidised and reduced solution. The absorbance of each point was then scaled to give the proportion of protein oxidised and the data from both titrations fitted to the equations below. Reported reduction potentials are quoted *vs* the Nernst hydrogen electrode (NHE).

3 $$y=\frac{10^{\frac{x-E1}{59}}+b}{1+10^{\frac{x-E1}{59}}+\;10^{\frac{E2-x}{59}}}$$

where *b* is proportional to the absorbance of the semiquinone species, *E*1 and *E*2 are the redox potentials of the semiquinone/hydroquinone and oxidised quinone/semiquinone transitions, respectively.

4 $$y=\frac{1}{1+\;10^{\frac{Em-x}{29.5}}}$$

where *Em* is the midpoint potential for the quinone/hydroquinone transition.

### Modelling and molecular dynamics

#### Structure preparation

Molecular dynamics simulations were based on the dimeric crystal structures in this work, modelled with the protein dimer with either oxidised FMN cofactor or reduced FMNH^-^ cofactor in both active sites. Input files for nitrofurantoin were generated with the Antechamber and Parmchk programmes of the AMBER 12 package [48] using atomic partial charges from Gaussian 09 [49] and parameters from the general AMBER force field (GAFF) [6]. Models for oxidised and reduced FMN were taken from previous work [50]. Final AMBER prep and frcmod files for nitrofurantoin used in this study are provided in the Supplementary Information. Hydrogen atoms were added to the amino acids of the protein models using the tleap programme of AMBER 12 according to physiological pH. The AMBER ff14SB force field [51] was applied to all amino acids. In each case, the system was solvated with a TIP3P water box with a minimum distance from protein surface to box edge of 10 Å, and sodium ions to neutralise the overall charge of the system.

#### Molecular dynamics simulations

All simulations were performed using the GROMACS 4.6.5 MD code [52]. ACEPYPE [53] was used to convert the topology from AMBER format to GROMACS format. Prior to the MD simulation, a molecular mechanics minimisation was performed on each structure, employing the steepest descent method, with a maximum force convergence criterion of 20 kJ mol^−1^ nm^−1^. Each simulation was equilibrated by 500 ps of constant pressure MD at 300 K and 1 bar. During minimisation and equilibration, position restraints of 1000 kJ mol^−1^ nm^−2^ were applied to the protein alpha carbons; aromatic carbons of nitrofurantoin and FMN ribityl carbons and phosphorus atoms. Unrestrained simulations, with co-ordinates saved every 10 ps, were run with temperature maintained at 300 K by a Nose–Hoover thermostat and pressure maintained at 1 atm with a Parrinello–Rahman barostat. The LINCS algorithm was applied to all bonds to allow a 2 fs timestep. A 10 Å cutoff was applied to electrostatic and van der Waals interactions, with the particle-mesh Ewald scheme, applied to long-range electrostatics. For each system, simulations were performed in an iterative fashion, as described previously [50]. The ptraj tool of AMBER 12 [48] and VMD 1.9.2 [54] were used for analysis, and UCSF Chimera 1.12rc was used for structure modification [47].

## Results and discussion

### Kinetics

**Figure 2.**
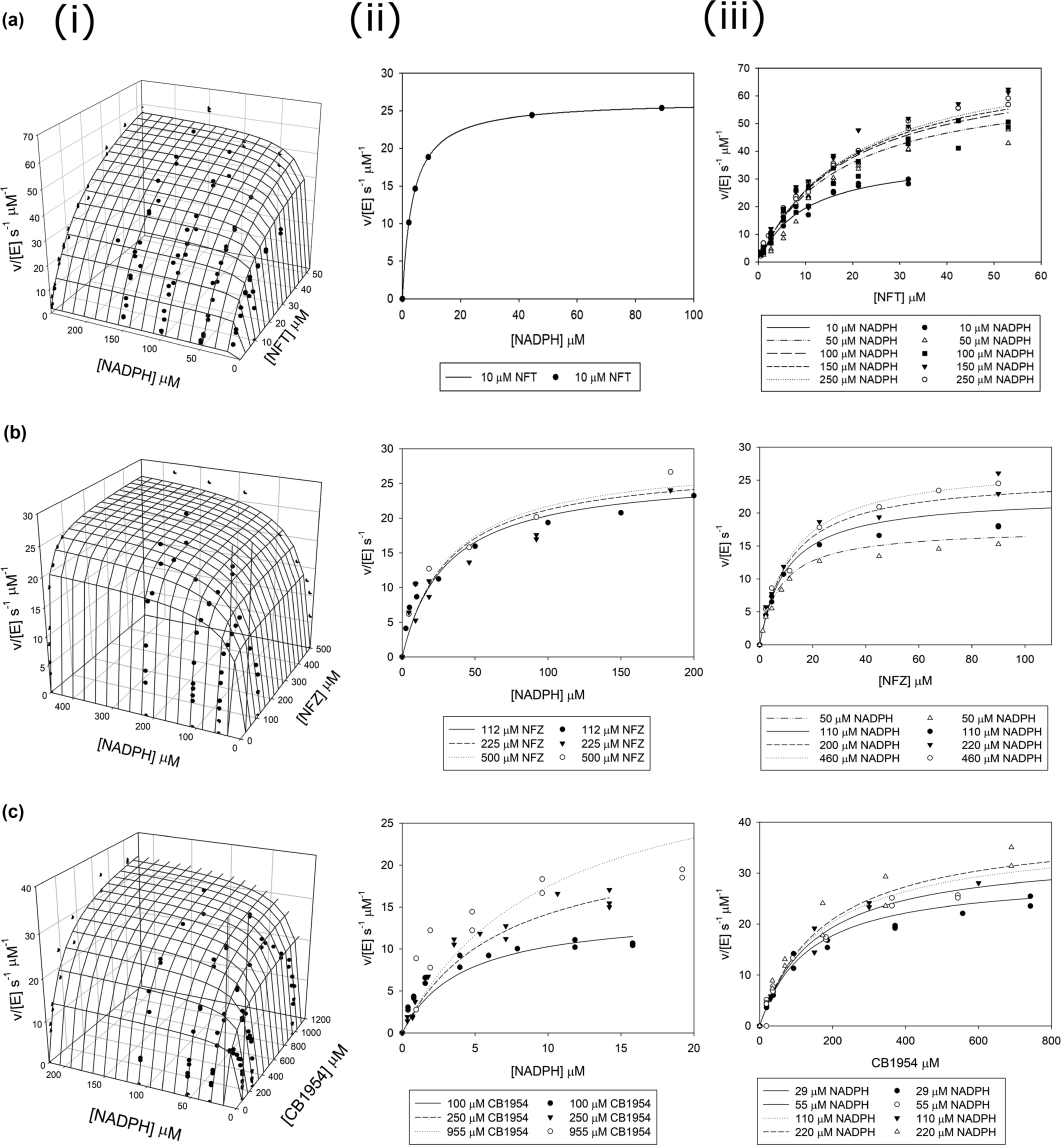
Steady-state kinetics of NfsA with nitroaromatic substrates. Steady-state kinetics of the reduction of (**a**) Nitrofurantoin, (**b**) Nitrofurazone, and (**c**) CB1945 by NADPH, catalysed by NfsA. Initial rates of reaction at different concentrations of NADPH and substrate were monitored. (i) The global, 3D fits of all the data; dots show the experimental rates and the mesh shows the fit to equation (1). (ii) Reactions done at different initial concentrations of NADPH at a series of constant substrate concentrations, the dots show the experimental points and the lines show the fits of the global rate constants. (iii) as (ii) but reactions were done at various concentrations of nitroaromatic substrate at different constant concentrations of NADPH. The reactions were measured in a 10 mM Tris pH 7.0 buffer and 4.5% DMSO, at 25°C; those with nitrofurantoin also contained 50 mM NaCl. (**a**) For nitrofurantoin, the lines are the simulations of equation (1) for *k*_cat_ 81 s^−1^, *K*_m_ nitrofurantoin 20.6 µM, and *K*_m_ NADPH 10.9 µM. (**b**) For nitrofurazone, the lines are the simulations for *k*_cat_ 29.6 s^−1^, *K*_m_ nitrofurazone 13.0 µM, and *K*_m_ NADPH 34.0 µM. (**c**) For CB1945 the lines are the simulations for *k*_cat_ 42.0 s^−1^, *K*_m_ CB1954 190 µM, and *K*_m_ NADPH 12.0 µM. The standard deviations and *P* statistics for the fits are given in Table 1.

*E. coli* NfsA was purified from an over-expressing strain of *E. coli*, as described previously [14]. The steady-state activity of the protein for nitrofurantoin, nitrofurazone and CB1954 was determined at a series of concentrations of NADPH and nitroaromatic substrate. The data were fitted to the global equation for a substituted enzyme reaction (eqn 1), to obtain the Michaelis parameters for these substrates (Figure 2, Table 1a). Steady-state activities were measured for the reduction of 1,4-benzoquinone and menadione by NADPH. The *K*_m_ values of these quinone substrates are very low, so the enzyme was only assayed at concentrations above the *K*_m_ of each substrate and cofactor (Table 1b).

The rates obtained show that the enzyme is active with all of these substrates, as shown previously [11,55]. This is the first report of global *K*_m_ values for substrate and NADPH cofactor on reduction of CB1954 and nitrofurantoin; previous assays have been reported only at a single concentration, 100 µM, of NADPH. Nonetheless, the *k*_cat_ values reported here are similar to the previous studies [11,55], as this concentration is well above the *K*_m_ for NADPH for these substrates. The *K*_m_ values for the nitroaromatic substrates are significantly lower than for NfsB [30], as are the *k*_cat_, hence NfsA is more active at low concentrations of nitroaromatics and is the first to be mutated in resistant *E. coli*; while NfsB is more active at high concentrations of nitroaromatics and is mutated later. The higher *k*_cat_/*K*_m_ values for nitroaromatics mean that NfsA is also better for cancer gene therapy and cell ablation studies, due to the low concentration of prodrugs that can be delivered into cells [14]. The substrate preference is also slightly different for NfsA and NfsB.

**Table 1.** Steady-state kinetic data for NfsA with various substrates at 10 mM Tris, pH 7.0, 25°C, in the presence of NADPH

Substrate	*k*_cat_ (s^−1^)	*P*	*K*_m_ (µM)	*P*	*k*_cat_/*K*_m_ (s^−1 ^µM^−1^)	*P*
*(a) Global fits, both substrates varied, nitroaromatic substrates*						
CB1954						
CB1954	42 ± 1	<0.0001	190 ± 20	<0.0001	0.23 ± 0.002	<0.0001
NADPH			12 ± 1	<0.0001	3.5 ± 0.3	<0.0001
Nitrofurazone						
Nitrofurazone	29.6 ± 0.9	<0.0001	13 ± 2	<0.001	2.3 ± 0.2	<0.0001
NADPH			34 ± 4	<0.001	0.88 ± 0.08	<0.0001
Nitrofurantoin (at 50 mM NaCl)						
Nitrofurantoin	81 ± 3	<0.0001	20.6 ± 1.6	<0.0001	3.9 ± 0.2	<0.0001
NADPH			10.9 ± 1.6	<0.0001	7.4 ± 1	<0.0001
**Substrate**	***k*_cat_ _app_ (s^−1^)**	***P***	***K*_m__app_ (µM)**	***P***	***k*_cat_/*K*_m_ (s^− 1^µM^−1^)**	***P***
*(b) Single fit, quinone substrates*						
Menadione						
Menadione (at 100 µM NADPH)	9.0 ± 0.4	<0.0001	11 ± 2	<0.0001	0.82 ± 0.08	<0.0001
NADPH (at 100 µM menadione)	12.8 ± 0.5	<0.0001	1.8 ± 0.3	<0.0001	7 ± 1	<0.0001
1,4 benzoquinone						
1,4 benzoquinone (at 50 µM NADPH)	24 ± 0.8	<0.0001	3 ± 0.4	0.0002	8 ± 0.8	<0.0001
NADPH (at 50 µM 1,4 benzoquinone)	31 ± 0.7	<0.0001	1.3 ± 0.2	0.002	23 ± 3	0.001

Rates for reactions where both substrate and cofactor were varied (**a**) were fitted to equation (1) whereas rates where only one substrate (or only the cofactor) was varied (**b**) were fitted to the simple Michaelis Menten equation, using nonlinear regression in Sigmaplot, with equal weighting of points, giving the statistics shown.

### Crystal structure with nitrofurantoin

The protein was crystallised in the presence and absence of several ligands (Supplementary Table S1). Screens based around previously published conditions for NfsA did not produce crystals [27], however, crystals were found using an in-house screen of small molecules and additives, based on the work of McPherson and Cudney [56] and optimised by sparse-matrix screening.

**Figure 3.**
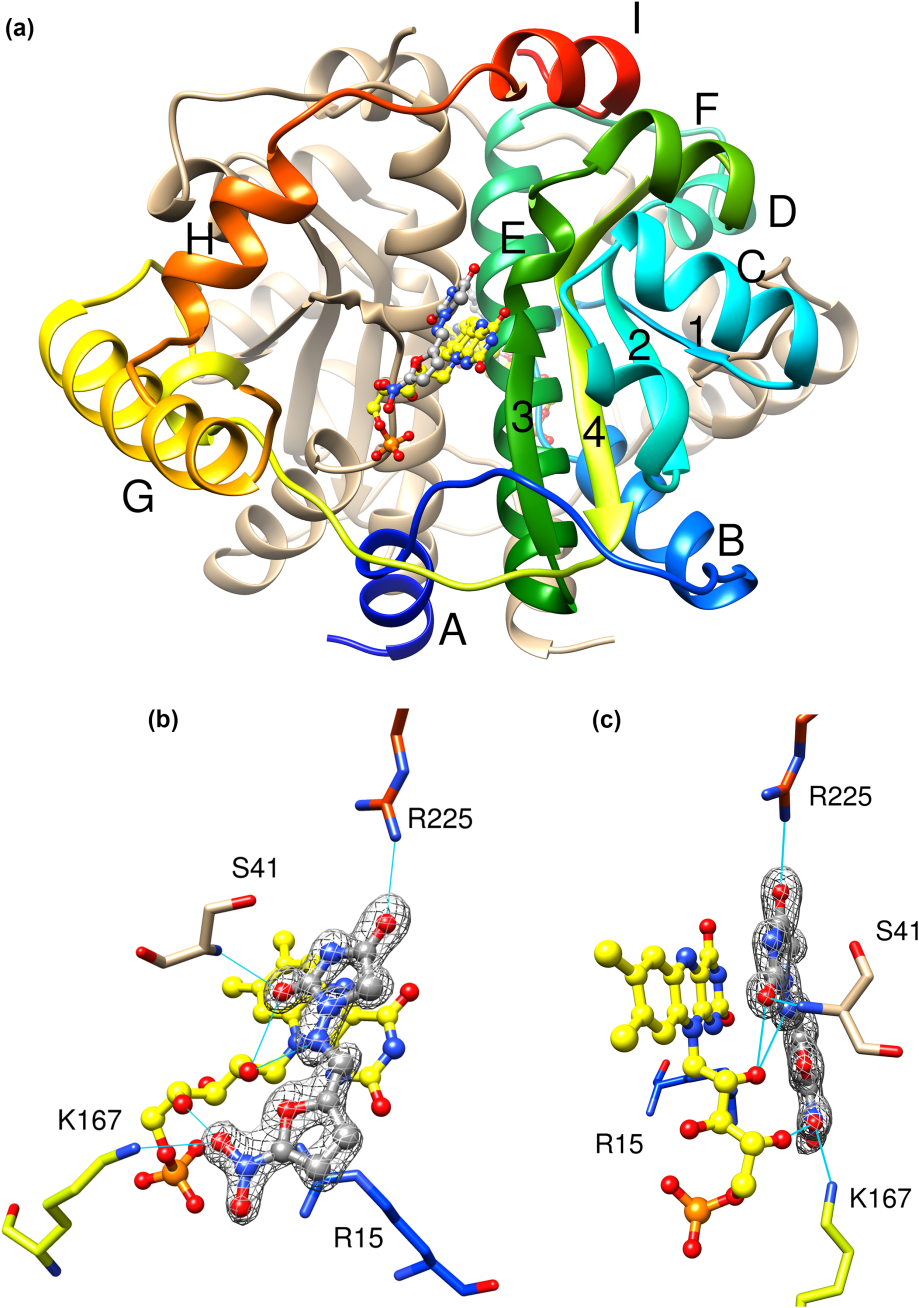
Crystal Structure of NfsA with nitrofurantoin. (**a**) Ribbon diagram of NfsA dimer, in the presence of nitrofurantoin. One subunit is in tan and the other is in rainbow colours blue to red from N- to C-terminus. The helices of the coloured subunit are labelled A-I, and the strands are numbered 1–4. The FMN cofactor is shown as ball and stick, with C atoms in yellow, N blue, oxygen red, and phosphorus orange. Nitrofurantoin is in ball and stick representation with C atoms in grey, and heteroatoms coloured as for FMN. (**b**,**c**) Two views of the nitrofurantoin binding site of NfsA. The FMN cofactor and nitrofurantoin are shown in ball and stick, coloured as in (**a**). The side chains that interact with nitrofurantoin are shown as sticks, labelled, with carbons atoms coloured as in the rainbow depiction of the backbone in (**a**), and heteroatoms coloured in CPK colours, as in (**a**). Cyan lines show the hydrogen bonding to the ligand. The mesh shows the electron density within a radius of 2 Å from the nitrofurantoin (level 0.44 e) at 1 σ.

The X-ray crystal structure of NfsA in presence of nitrofurantoin, at 1.09 Å resolution, is shown in Figure 3a. The structure of NfsA in the absence of ligands was determined previously by Kobori et al. [27], in a different space group with a dimer in the ASU, but with similar crystal contacts. As shown previously for the free protein [27], the protein is dimeric with a mixed alpha/beta structure (Figure 3a). Each subunit contains two domains, the core domain of the four beta strands surrounded by alpha helices, and an excursion domain, residues 165–210 containing one long helix, G, a shorter helix H and long loops. The two monomers have extensive contact. The major dimer interface is at helix E, the longest alpha helix in each subunit (residues 100–120), with the FMN cofactors on either side of these helices. The two FMN cofactors are ∼27 Å apart and each sits near the hydrophobic base of a cavity surrounded by positively charged groups. Each FMN cofactor contacts both subunits, via hydrogen bonds and extensive van der Waals contacts.

The nitrofurantoin binds parallel to the FMN ring and is in van der Waals contact with the whole of the ring and the ribitol chain, but, surprisingly, the imidazolidine ring of nitrofurantoin, rather than the nitro group to be reduced, is stacked above the central ring of the FMN (Figure 3b,c). A network of hydrogen bonds is formed between the ligand, the ribityl chain of the FMN and both subunits of the protein, involving Arg 15, Lys 167 and Arg 225, (from one subunit) and Ser 41B (where B denotes the other subunit) as shown.

There is little difference in the conformation of NfsA with and without nitrofurantoin, apart from at a surface loop, residues 203–211, between helices G and H (see Figure 8). This has high B factors in both structures and is likely to be poorly modelled in both. The RMSD between the two structures for all the backbone atoms is 0.28 Å.

### Nitrofurantoin mechanism

If there were direct hydride transfer between the FMN and the ligand, the nitro group of the furan ring should be directly below the N5 of the FMN to allow reduction. The reversed orientation of the rings and the FMN seen here with NfsA and nitrofurantoin was also found in our crystal structure of nitrofurazone with NfsB [30]. It has also been found in Pentaerythritol tetranitrate reductase with steroids [57], in Xenobiotic reductase A with coumarin derivatives [58], and in *E. cloacae* NR with nitrobenzoate [32]. All these structures are of dead-end complexes, with oxidised enzyme. We and others have suggested that the orientation of the substrate may change in the reduced enzyme, where the FMN is negatively charged and has a different electron distribution. It is also possible that this is a very stable but non-reactive complex which competes with the reactive orientation with the reverse orientation. A reactive orientation of nitrofurazone has been seen with Azoreductase [59].

Alternatively, it has been suggested that nitroreduction may occur by an initial electron transfer from FMN, rather than hydride transfer. This would be quickly followed by a proton transfer from bound water, transfer of the second electron from FMN with a second proton almost simultaneously. In such a mechanism, only the electron orbitals of the FMN and substrate need to overlap, and so the orientation seen in the crystal structure could be productive [60,61]. Initial MD simulations of *E. coli* NfsB with CB1954 [60], and of *E. cloacae* NR with nitrobenzene [62] suggested that this e-..H+..e- the mechanism was feasible. This idea was disputed after kinetic studies of *E. cloacae* NR, with deuterated NADH [32] showed small kinetic H/D isotope effects on the reduction in nitrobenzoate. However, the small size of the effect is consistent with a secondary isotope effect, from a change in hybridisation of the NH group on reduction, as well as from a small primary isotope effect from direct hydride transfer, so the alternative mechanism has not been disproved.

**Figure 4.**
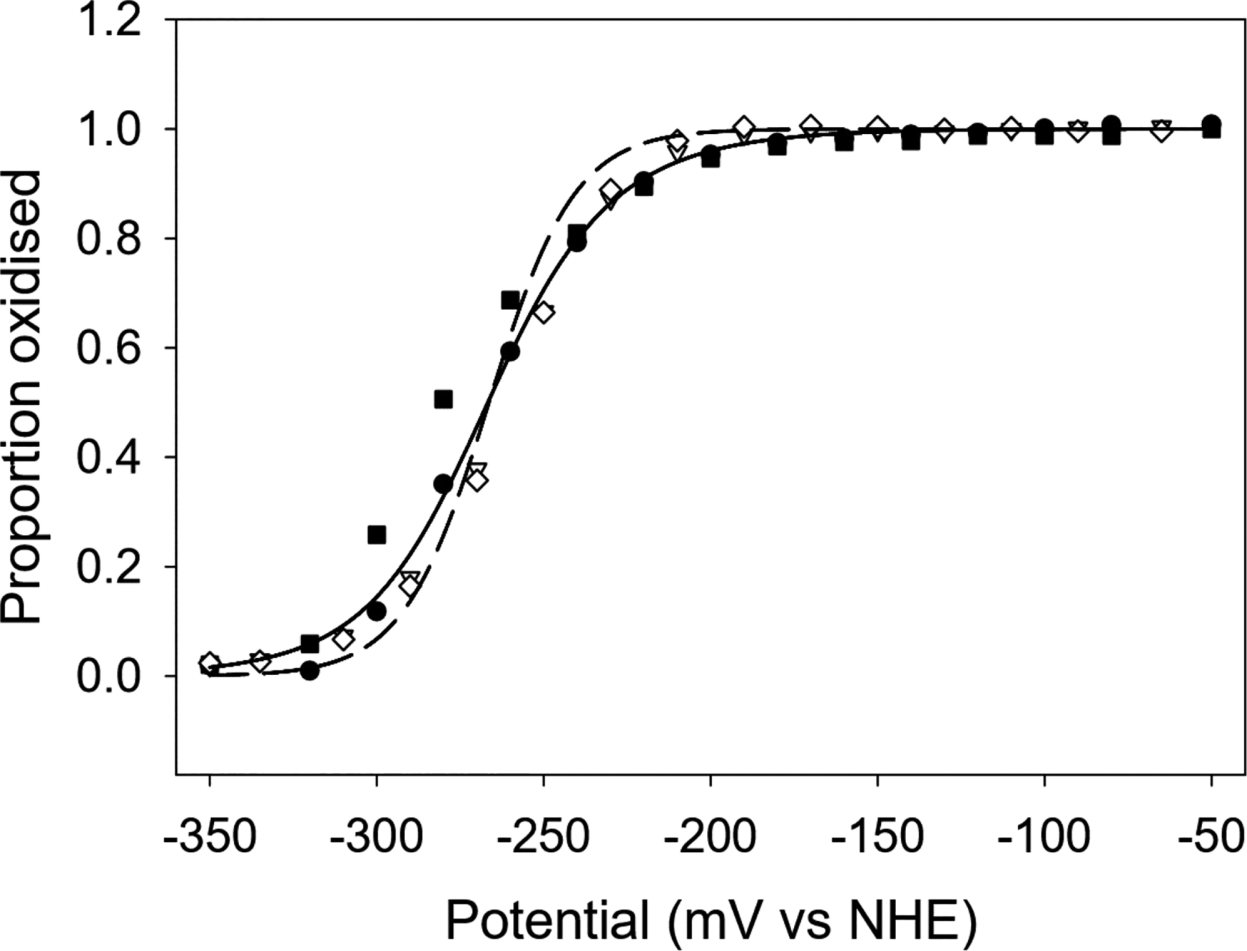
Potential titration of NfsA. Symbols show two redox titrations, on two aliquots of the same enzyme preparation. Titrations were performed with 80 μM NfsA in 50 mM phosphate buffer, pH 7.5, 500 mM KCl, 10% glycerol, in the absence of redox mediators. Black symbols, oxidation cycles — square experiment 1, circle experiment 2. White symbols — reduction cycles, triangles, experiment 1, diamonds experiment 2. Dashed line — fit of data to a concerted two-electron transfer, with midpoint potential −264 mV; solid line — fit of data to two single-electron steps with potentials −272 mV and −268 mV, respectively.

To test whether the single electron, semiquinone state is stable, we measured the redox potential of the free NfsA protein. The data fits two single electron transfer steps with *E*1 = −272 ± 7 V and *E*2 = −268 ± 10 V statistically much better than to a single two-electron transfer (Figure 4); however, the difference the in two redox potentials *E*1 and *E*2 is less than the standard deviation for these measurements. As both potentials are similar and as *E*1 is more negative than *E*2, little semiquinone is likely to form with the free enzyme and the proportion of this in the titration is poorly determined. This finding is similar to that for NR from *E. cloacae* [63] which showed a simultaneous two-electron reduction in redox titrations. While the redox potentials may change in the presence of substrate, these data are consistent with these reductases being oxygen insensitive.

### Molecular dynamics simulations

**Figure 5.**
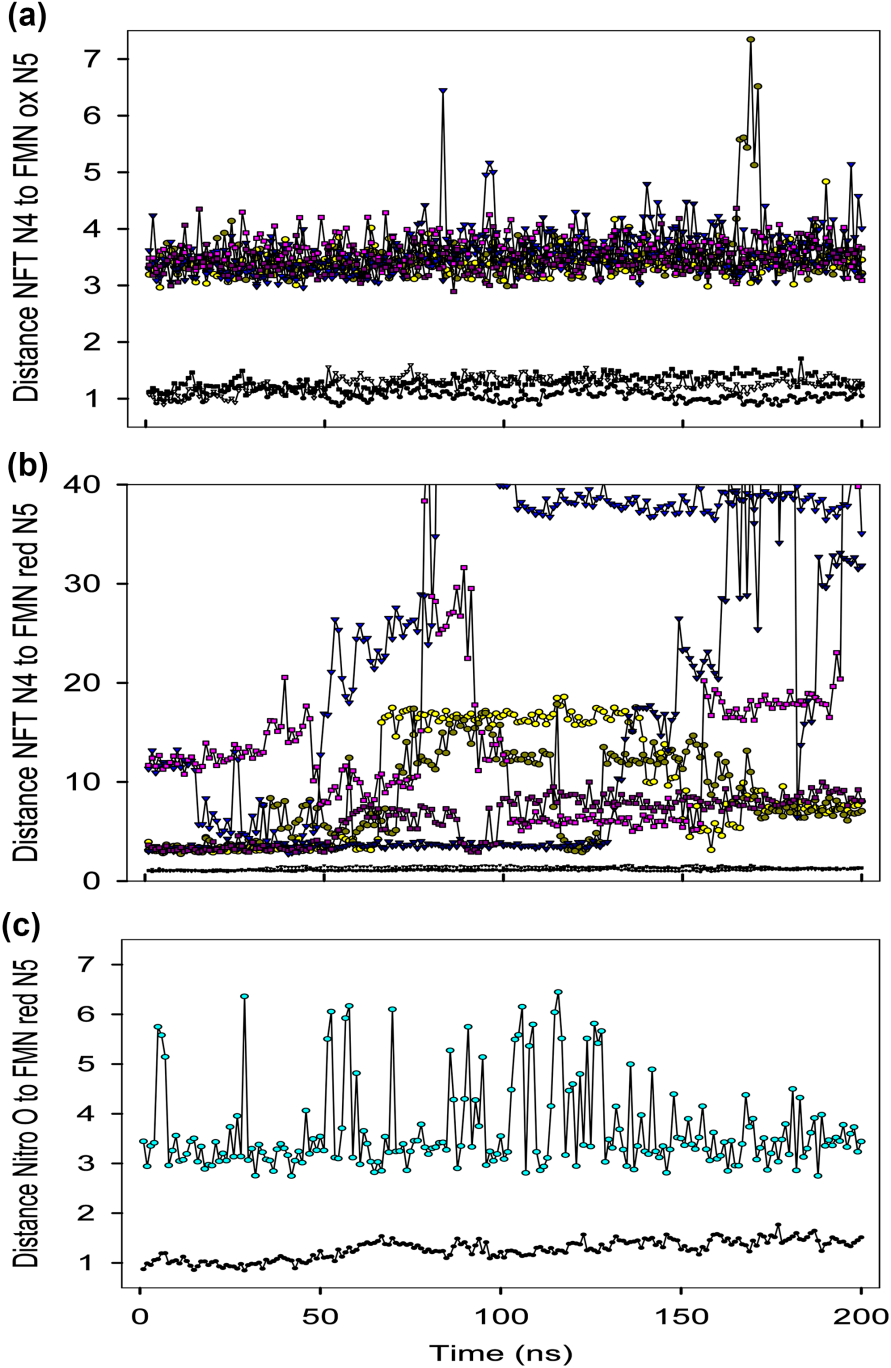
MD simulations of Nitrofurantoin bound to NfsA. (**a**) Plot of the distance between the nitrofurantoin N4 amide and FMN N5 in oxidised NfsA. Three separate molecular dynamics simulations of nitrofurantoin in NfsA were run based on the crystal structure of the dimeric complex, with nitrofurantoin in both active sites. The RMSD of the protein backbone from the initial structure is shown with black markers, circles for run 1, triangles for run 2, and squares for run 3. The distances of the N4 amide group of nitrofurantoin to the FMN N5 in each run are shown in yellow/dark yellow (for site 1 and site 2, respectively) for run 1, in blue/dark blue for run 2, and in pink/dark pink for run 3. All distances are in Ångstrom. (**b**) Centre- as in figure a, but now with NfsA reduced. (**c**) Distance of the nitro oxygen of nitrofurantoin to the FMN N5 in a simulation with reduced dimeric NfsA and nitrofurantoin bound in the opposite orientation to that in the crystal structure, in a single site, as in (**a**,**b**), with the nitro oxygen close to the FMN N5. The RMSD of the protein backbone from the initial structure is shown with black circles, the distance of the nitro oxygen to FMNH_2_ N5 is shown in cyan.

Molecular dynamics (MD) simulations of our structure of nitrofurantoin with oxidised NfsA dimer show that the orientation of the ligand in the crystal structure is stable over 200 ns, with both sites simultaneously occupied by nitrofurantoin (Figure 5a, and Supplementary Table S2). When the simulations were repeated with reduced NfsA, with FMNH^-^ in the active site, in all cases neither of the two nitrofurantoin ligands remained stably bound in the active site (Figure 5b, and Supplementary Table S2). These simulations show that the ligand binds differently to oxidised and reduced enzyme. Recent MD simulations of *E. cloacae* NR with p-nitrobenzoate, based on the crystal structure [32], with improved force field parameters for the ligands and cofactors, show an analogous difference in the orientation of the ligand in the oxidised enzyme compared with the reduced enzyme [50].

**Figure 6.**
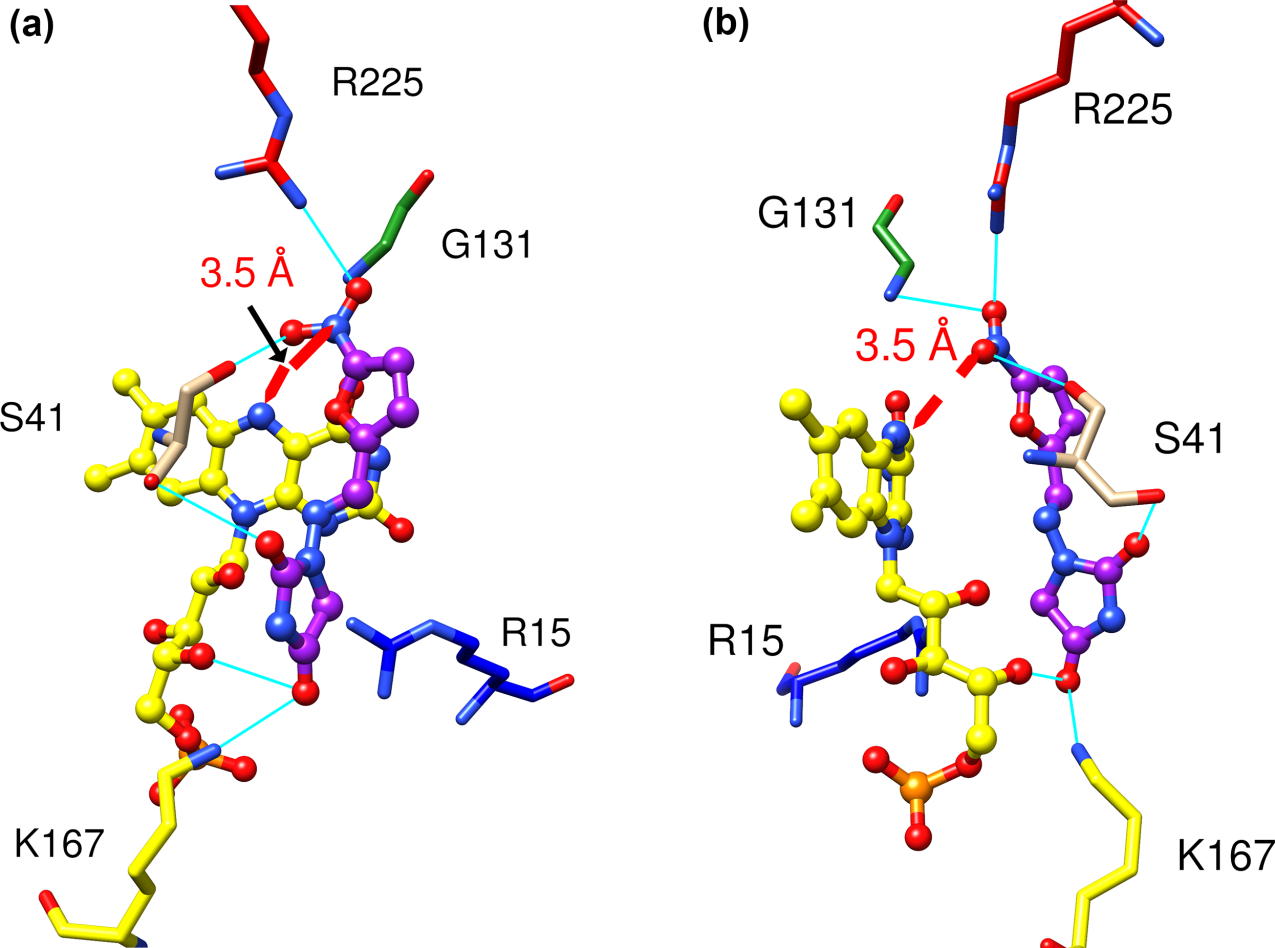
Model of nitrofurantoin bound to reduced NfsA. (**a**,**b**) Two views of nitrofurantoin modelled bound to reduced NfsA. The FMNH^-^ cofactor is shown in ball and stick, coloured as in Figure 3. Nitrofurantoin is shown in ball and stick representation with the carbon atoms in purple and the heteroatoms coloured as for FMN. The side chains that interact with the nitrofurantoin are shown as sticks, labelled, with carbons atoms coloured as the ribbon in Figure 3a, and heteroatoms coloured as in Figure 3a. Cyan lines show the hydrogen bonding to the ligand. The red arrow shows the distance between the N5 atom of FMN and one nitro oxygen atom of the ligand, appropriate for direct hydride transfer.

The MD simulations, together with the potentiometric titrations, indicate that the mechanism of reduction is likely to be direct hydride transfer from the N5 of FMNH^−^ to the nitro oxygen. A largely direct hydride transfer mechanism for NfsA was also suggested by Valiauga et al. [55] in studies of reduction in quinones, and tertyl. We have, therefore, modelled a complex of reduced enzyme with the nitro oxygen of nitrofurantoin 3.6 ± 0.8 Å from the N5 of FMNH^-^ (Figure 6a,b), the appropriate distance for direct hydride transfer. In MD simulations, over 200 ns, this model of the fully reduced enzyme dimer with nitrofurantoin in a single site, has good binding enthalpy (maximum observed −21.9 ± 0.3 kcal mol^−1^), with several hydrogen bonds from the protein to the nitro group and the furan ring (Figure 6), but the distance between the nitro oxygen and the N5 of FMNH^-^ fluctuates within the simulation (Figure 5c, Supplementary Table S2).

### Crystal structures with 1,4-benzoquinone and hydroquinone

**Figure 7.**
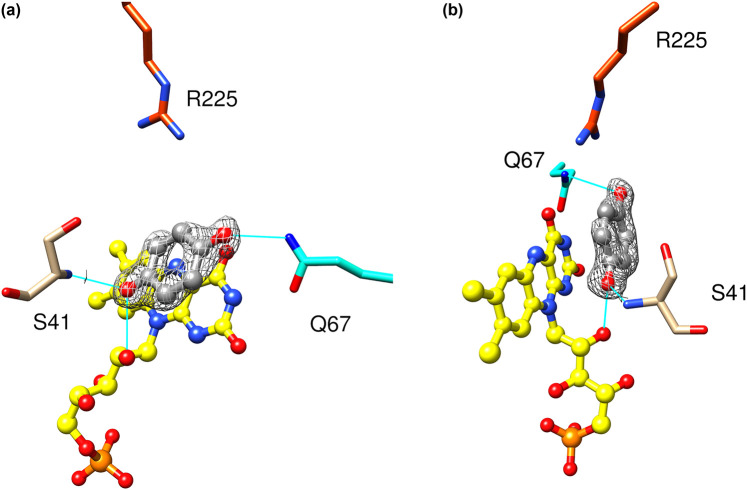
Structure of NfsA with bound hydroquinone. (**a**,**b**) Two views of hydroquinone bound to NfsA. The FMN cofactor is shown in ball and stick, coloured as in Figure 3. Hydroquinone is shown in ball and stick representation with the carbon atoms in grey and the oxygen atoms in red. The side chains that interact with the ligand are shown as sticks, labelled, with carbons atoms coloured as the ribbon Figure 3a, and heteroatoms coloured as in Figure 3a. Cyan lines show the hydrogen bonding to the ligand. The mesh shows the electron density within a radius of 2 Å from the hydroquinone (level 0.72 e) at 1.5 σ.

NfsA reduces quinones as well as nitroaromatics, and these have rather different geometries. To examine the binding and orientation of quinone substrates, we crystallised the protein in the presence 1,4-benzoquinone and hydroquinone. These are both symmetrical, 1,4-benzoquinone forming a dead-end complex with oxidised NfsA while hydroquinone is the product complex of the reaction. The structure with 1,4-benzoquinone was determined at 1.70 Å resolution, whereas that with hydroquinone was at 1.25 Å resolution. The two structures were almost identical and showed no change in the backbone of the protein from that in the absence of ligand or with nitrofurantoin. Both hydroquinone and 1,4-benzoquinone showed similar density in the active site, which is slightly smeared, with high B factors for the ligand, suggesting motion of the benzene ring in a plane parallel to the flavin ring. There are extensive van der Waals interactions or *π* stacking between the ligands and the FMN cofactor. One oxygen group of the ligand interacts with the O2′ hydroxyl of the FMN and the backbone NH of Ser 41 whilst the other may interact with the amine side chain of Gln 67 and is close to Arg 225 (Figure 7a,b). The carbon of the latter is 3.7 Å from the N5 group of the FMN, in a good position for direct hydride transfer.

NfsA contains five cysteines at positions 9,45 80, 90 and 156. In the 1,4-benzoquinone complex, Cys 90 showed an additional quinol ring within covalent bonding distance to the SH side chain, suggesting that this cysteine reacted at the two positions of the quinone to form a S-cysteinyl, hydroquinone adduct.

### FMN as ligand

**Figure 8.**
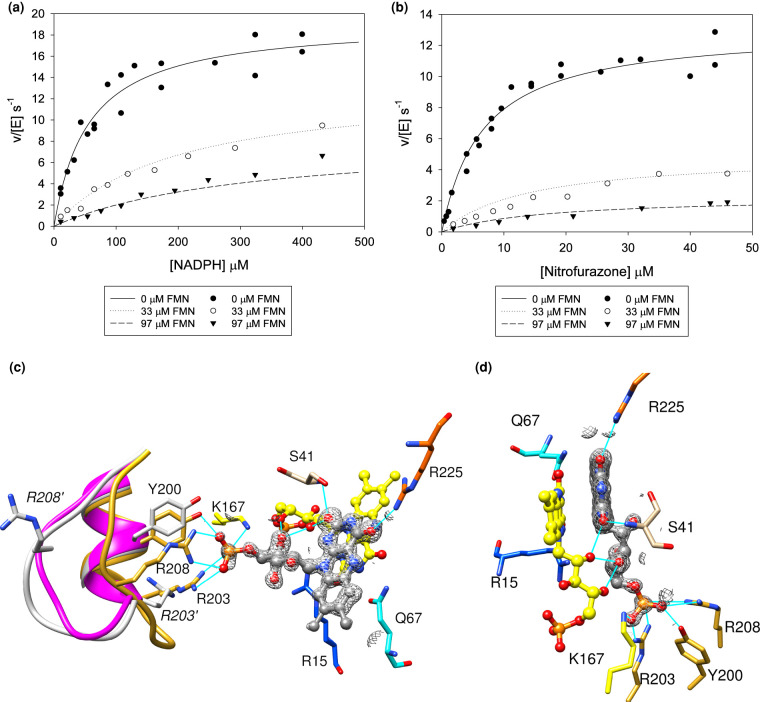
Kinetics and structure of NfsA with FM. (**a**,**b**) Steady-state kinetics of NfsA with nitrofurazone in the presence or absence of FMN. (**a**), reactions done with 99 µM nitrofurazone, varying NADPH. (**b**), reactions done with 97 µM NADPH, varying nitrofurazone. All reactions were done in a 10 mM Tris pH 7.0 buffer containing 50 mM NaCl and 4.5% DMSO, at 25°C. The symbols show the measured rates and the lines show the simulated Michaelis Menten curves for mixed inhibition, with *K*_m_ NADPH 62 µM, *K*_m_ nitrofurazone 11 µM, *k*_cat_ 21.4 s^−1^, *K*_i_ NADPH 8 µM, and *K*_i_ nitrofurazone 7 µM. The standard errors in the fitting parameters and the P statistics for the data are given in Supplementary Table S3. (**c**) The structure of a second FMN bound to NfsA. The FMN ligand is shown in ball and stick representation with the carbon atoms in grey and the heteroatoms coloured as in Figure 3a. The FMN cofactor is coloured as in Figure 3a. The backbone of residues 198–210 and carbon atoms of selected side chains of that region are coloured in gold for the FMN-bound structure, grey for the nitrofurantoin-bound structure and purple for the structure in the absence of ligand from 1F5V [27]. Side chains of residues R203 and R208 from the nitrofurantoin-bound structure are labelled in italics and given a prime symbol. All other side chains, in normal font, are from the FNM-bound structure and are coloured as in Figure 3a, with the heteroatoms of the side chains coloured blue for nitrogen and red for oxygen. The mesh shows the electron density within 2 Å of the FMN ligand at 0.7 sigma, (0.092 e). Cyan lines show the hydrogen bonds between the protein, the FMN cofactor, and the FMN ligand. (**d**) A second view of the structure shown in (**c**), with the same colouring.

In our initial crystal trials with hydroquinone, we obtained a structure with second FMN as a ligand in the active site of the protein, as well as the FMN cofactor. This arose from the addition of excess FMN to the protein solution during protein purification, and in subsequent experiments, the excess FMN was removed by dialysis of the protein. While FMN is not a substrate for NfsA, NfsA is homologous to Frp, the flavin oxidoreductase from *Vibrio harveyi*, and mutation of single residue, E99G, has been shown to convert NfsA into an active FMNase [64]. Our kinetic studies show that FMN binds to both oxidised and reduced forms of the enzyme, giving *K*_i_ ∼ 8 µM with respect to both nitrofurazone and NADPH (Figure 8a,b, Supplementary Table S3). Inhibition of the rate of reduction in nitrofurazone by NfsA in the presence FMN was also observed by Zenno et al. [11] but the dissociation constant was not measured. In contrast, the activity of NfsB was found not to be affected by FMN [11].

In the crystal structure, determined at 1.03 Å resolution, the isoalloxazine ring of the inhibitor FMN is parallel to that of the FMN cofactor but displaced so that only two of the three rings overlap, and rotated 180° about the C1′-N10 bond (Figure 8c,d). Thus the pyrimidine dione ring is above the central ring of the prosthetic group and the central ring of the FMN ligand is above the dimethylbenzene ring of the cofactor. The electron density of the dimethyl benzene ring of the inhibitor is weaker than that of its pyrimidine dione ring, suggesting that there may be some motion of the FMN about the N3 nitrogen. The FMN inhibitor forms hydrogen bonds to the cofactor FMN, to the backbone of Ser 41B, and the guanidinium group of Arg 225. One Me group is close to Gly 65, while Gln 67 is close to the N5.

The phosphate group of the ligand FMN is bonded to the guanidinium groups of Arg 203 and Arg 208 and to Tyr 200 and Lys167. This binding causes the backbone of residues 203–211 in the mobile loop between helices G and H to change conformation from that in all the other liganded structures, as well as that of the free protein from Kobori et al. (Figure 8c). In particular, the Cα atom of Arg 208 moves 7 Å, while, at the tip of the loop, the Cα atom of Asn 206 moves 11 Å. The B factors of the residues in this altered loop are lower than this loop in the other structures and are now similar to those of the rest of the protein.

In this structure, the phosphate is modelled at only 50% occupancy, and the co-ordinating residues in the structure are modelled in two orientations, with 50% of the loop in the bound conformation and 50% in the same conformation as in the unliganded structures. In this complex, the protein crystals had been soaked with hydroquinone, and two surface cysteines, Cys 90 and Cys9, appeared to be covalently bonded to quinone.

NfsA is specific for NADPH over NADH, and the conformational change of residues 203–211 seen for FMN is also likely to occur with NADPH to form a similar phosphate-binding pocket, involving the same residues. Kobori et al. [27] also postulated that this loop was involved in NADPH binding and mutated the two arginine residues. R203A increased the *K*_mapp_ of NADPH ∼33 fold but had little effect on the *K*_mapp_ of NFZ, implying its involvement in binding NADPH but not nitrofurazone; however, the mutation R208A had little effect. We propose that in the R208A mutant the interaction of the phosphate group with R208 is replaced by one with R209, the adjacent residue on the mobile loop. Residue 203 is conserved across NfsA homologues, and its presence correlates with NADH/NADPH preference, [9], R or *K* is often seen at position 208 but the mobile loop is not present in NfsB, which does not discriminate between NADH and NADPH. In FRP, mutations of K167 and R15 which both bind to the 2′ phosphate group in our structure, were also shown to affect NADPH binding while N134A, R133A and R225A had little effect [65].

While FMN is an inhibitor of NfsA, a single amino acid substitution E99G causes it to have similar activity as FRP, the Flavin oxidoreductase from *V. harveyi* [64]. Glu 99 forms hydrogen bonds to both Arg 133 and Arg 225 across the dimer interface. In the homologous protein FRP, the mutation E99K destabilises the dimer interface, increasing the dissociation constant of the dimer 44-fold [66]. In NfsA, the E99G mutation would remove the backbone brace of Glu 99 to the two arginine residues and so may allow Arg 225 to move and let the ligand FMN to be positioned over the N5 position of the cofactor, hence becoming a substrate.

## Conclusions

We have determined the structures of oxidised NfsA bound to nitrofurantoin, to 1.4-benzoquinone, hydroquinone and to a second FMN. The proteins have similar backbone conformations in all complexes apart from a mobile loop, residues 202–211, that on binding FMN forms part of a phosphate-binding pocket. This phosphate-binding pocket containing residues Arg 203, Arg 208, Tyr 200 and Lys 167 is likely to also form with NADPH, making NfsA specific for NADPH over NADH. The ligands bound to NfsA all stack with the FMN and interact with the 2′OH ribitol group of FMN and the backbone NH of Ser 41B. Most also interact with Arg 225. Arg 203 and Arg 225 were noted as being conserved in 61% of the NfsA subfamily of 2 299 sequences [9,10] but not in other subfamilies.

The redox and molecular dynamics studies suggest that the kinetic mechanism of NfsA is most likely to be direct hydride transfer to and from the flavin. 1,4-benzoquinone and hydroquinone bind similarly to the active site of NfsA, in an appropriate orientation and distance to react with the N5 position of FMN. In contrast, the asymmetrical substrate nitrofurantoin, which has a higher *K*_m_ than benzoquinone, binds the oxidised enzyme in a nonproductive orientation. There are no changes in the protein backbone, but the antibiotic changes orientation when the flavin cofactor is reduced. Analogous non-productive binding of substrates to oxidised enzyme has been found in crystal structures of several other flavoproteins. Such non-productive binding of substrates to oxidised flavoproteins may be general and, as there is no effect on the protein backbone, must be governed largely by the charge distribution of the flavin cofactor within the protein. We have made a model of nitrofurantoin bound to reduced NfsA, in a viable hydride transfer orientation, to help in the design of improved nitrofuran antibiotics.

## Data Availability

The crystallographic data have been deposited in the Protein Data Bank with accession codes 7NB9 for the nitrofurantoin-bound structure, 7NNX for the 1,4-benzoquinone structure, 7NMP for the hydroquinone structure and 7NIY for the FMN-bound structure. The model of the reduced enzyme bound to nitrofurantoin has been deposited into the modelarchive (modelarchive.org.doi) as project ma-9z55z.

## Open Access Statement

Open access for this article was enabled by the participation of University of Birmingham in an all-inclusive Read & Publish pilot with Portland Press and the Biochemical Society under a transformative agreement with JISC.

## Abbreviations

ASU: asymmetric unit
MD: molecular dynamics
NFT: nitrofurantoin
NFZ: nitrofurazone
NMP: N-methyl pyrolidone
PEG: polyethylene glycol
RMSD: root-mean-square deviation
