# Supplementary material for: The structures of E. coli NfsA bound to the antibiotic nitrofurantoin; to 1,4-benzoquinone and to FMN
Source: Biochem J. 2021 Jul 9;478(13):2601–17. doi: 10.1042/BCJ20210160 (PMC8286842; doi:10.1042/BCJ20210160)
Supplement: Supplementary Tables S1-S3 [file BCJ-478-2601-s1.pdf]

	**NFT**	**1,4 Benzoquinone**	**1,4 Hydroquinone**	**FMN**
PDB code	7NB9	7NNX	7NMP	7NIY
Source	ESRF-ID 23-1	Rigaku	Rigaku	ESRF-ID14-1
Data collection				
Unit cell (Å):	91.40, 51.86, 64.58	91.77, 51.96, 64.69	91.98, 52.02, 64.77	91.91, 52.00, 64.69
Unit cell (°)	90.00, 134.1, 90.00	90.00, 133.92, 90.00	90.00, 134.16, 90.00	90.00, 133.78, 90.00
Space group:	C 1 2 1	C 1 2 1	C 1 2 1	C 1 2 1
Resolution range (Å):	45.69-1.09 (1.09-1.15)	46.59-1.70 (1.70-1.79)	46.03-1.25 (1.25-1.35)	32.34 -1.03 (1.03-1.06)
Wavelength (Å):	1.00	1.54	1.54	0.934
Number of reflections	283,824 (21,970)	273,625 (16,275)	379,094 (25,991)	844,480 (25,188)
Unique reflections:	82,863 (9425)	23,824 (3,102)	56,273 (3,133)	99,892
Multiplicity	3.4 (2.33)	11.4 (5.3)	6.4 (3.5)	8.0 (3.4)
Completeness (%):	91.4 (64.5)	98.2 (88.7)	97.6 (84.9)	96.7 (92.3)
<*I*/σ*I*> overall	19.4 (3.7)	24.8 (4.6)	19.2 (2.4)	27.9 (2.1)
R_{Sym} [1] (%)	3.7 (22)	9.1 (35)	5.2 (55)	4.4 (58)
Refinement and quality				
R_{work}[2]:	0.110	0.153	0.124	0.108
R_{free}[3]:	0.130	0.177	0.156	0.121
RMSD bond length (Å)	0.015	0.008	0.010	0.015
RMSD bond angles (°)	1.839	1.260	1.496	1.752
RMSD Chirality	0.104	0.08	0.101	0.096
RMSD planarity	0.017	0.06	0.009	0.012
Fo Fc correlation	0.98	0.96	0.98	0.98
Number of heavy atoms	2,396	2,364	2,410	2,644
Number of protein heavy atoms	1887	1875	1875	1903

Number of ligand heavy atoms	52	64	56	81
Number of waters	297	258	287	295
Average B factors (Å^2)	18	17	17	11
Protein	15	14	14	8
Ligands:	13<br>NFT 16<br>FMN 9	18<br>PLQ 29<br>FMN 8	17<br>HQE 24<br>FMN 8	11<br>FMN 13<br>FMN 4
Waters:	32	27	30	25
Ramachandran plot				
Favoured regions (%)	95	97	97	97
Allowed regions (%)	5	3	3	3
Disallowed regions (%)	0	0	0	0

- The numbers in parentheses represent statistics in the highest resolution shell.
- *1.* $R_{sym} = \Sigma|I_i - \langle I \rangle|/\Sigma|I_i|$ where I_i is the intensity of the *i*th measurement, and $\langle I \rangle$ is the mean intensity for that reflection;
- 2.$R_{work} = \sum||F_o| - |F_c|/\sum|F_o|$, where F_o and F_c are the observed and calculated structure factors for data used for refinement, respectively.
- *3.* $R_{free} = \sum||F_o| - |F_c|/\sum|F_o|$ for 5% of the data not used at any stage of structural refinement.

Supplementary Table 1: Crystallographic data collection and refinement statistics for NfsA bound to ligands.

	Run 1		Run 2		Run 3	
**Active site**	**Site 1**	**Site 2**	**Site 1**	**Site 2**	**Site 1**	**Site 2**
**NFT in crystal structure orientation, oxidized NfsA**						
NFT N4 to FMN N5 (Å)	3.4 ± 0.2	3.5 ± 0.7	3.6 ± 0.5	3.5 ± 0.3	3.6 ± 0.3	3.4 ± 0.2
Cα RMSD (Å)	1.1 ± 0.1		1.3 ± 0.1		1.3 ± 0.1	
Binding Enthalpy (kcal/mol)	−23.0 ± 0.4	−24.3 ± 0.4	−17.2 ± 0.4	−17.0 ± 0.4	−18.0 ± 0.3	−18.1 ± 0.3
**NFT in crystal structure orientation, reduced NfsA**						
NFT N4 to FMN N5 (Å)	10 ± 6	9 ± 4	30 ± 10	10 ± 10	14 ± 9	6 ± 2
Cα RMSD (Å)	1.2 ± 0.1		1.4 ± 0.2		1.2 ± 0.1	
Binding Enthalpy (kcal/mol)	Not bound	Not bound	Not bound	Not bound	Not bound	Not bound
**NFT in hydride transfer orientation, reduced NfsA**						
NFT nitro O to FMN N5 (Å)	3.6 ± 0.8					
Cα RMSD (Å)	1.3 ± 0.2					
Binding Enthalpy (kcal/mol)	−21.9 ± 0.3 (best observed)					

Supplementary Table 2. Numerical averages and standard deviations of selected distances and energies for molecular dynamics simulations over 200 ns of nitrofurantoin bound in different orientations to oxidized and reduced NfsA. Binding enthalpies are measured over the final 5 ns of the simulation unless indicated otherwise.

Inhibitor	Substrate	k_{cat} (s^{-1})	P	K_m (μM)	P	k_{cat}/K_m (s^{-1} $μM^{-1}$)	P	K_i (μM)	P
**FMN**	Nitrofurazone (at 97 μM NADPH)	21.4 ± 0.7	<0.001	11 ± 1	<0.0001	2.0 ± 0.2	<0.0001	7 ± 2	0.002
	NADPH (at 99 μM NFZ)			62 ± 6	<0.0001	0.34 ± 0.02	<0.0001	8 ± 1	<0.0001

Supplementary Table 3: Steady-state kinetic data for NfsA with nitrofurazone and NADPH in 10 mM Tris HCl pH 7.0, 50 mM NaCl, 4.5 % DMSO, 25 °C in the presence of FMN. Initial reaction rates were measured for a range of concentrations of one substrate in the presence of a fixed concentration of the second substrate, in the presence or absence of inhibitor (at 2 concentrations). Rates were fitted to Equation 2, using non-linear regression in Sigmaplot, with equal weighting of points, giving the statistics shown.

AMBER force field prep and frcmod files for ligands and cofactors

Nitrofurantoin (NFT.prep)

```
    0    0    2

This is a remark line
molecule.res
NFT   INT  0
CORRECT     OMIT DU   BEG
  0.0000
   1  DUMM  DU    M    0  -1  -2     0.000      .0        .0      .00000
   2  DUMM  DU    M    1   0  -1     1.449      .0        .0      .00000
   3  DUMM  DU    M    2   1   0     1.523   111.21       .0      .00000
   4  O5    o     M    3   2   1     1.540   111.208  -180.000 -0.502500
   5  C8    c     M    4   3   2     1.203    68.898     0.000  0.555300
   6  N4    n     M    5   4   3     1.408   126.807    -0.000 -0.504600
   7  H1    hn    E    6   5   4     1.011   121.419     0.000  0.377500
   8  C1    c     M    6   5   4     1.380   114.628   180.000  0.504300
   9  O1    o     E    8   6   5     1.209   127.651   180.000 -0.497300
  10  C2    c3    M    8   6   5     1.533   105.419    -0.000 -0.077200
  11  H2    h1    E   10   8   6     1.097   110.280   119.898  0.100100
  12  H3    h1    E   10   8   6     1.097   110.307  -119.970  0.100100
  13  N1    n     M   10   8   6     1.460   102.834    -0.000  0.152000
  14  N2    n2    M   13  10   8     1.343   128.029   180.000 -0.398500
  15  C3    ce    M   14  13  10     1.290   118.693     0.000  0.166000
  16  H4    h4    E   15  14  13     1.092   123.912     0.000  0.064800
  17  C4    cc    M   15  14  13     1.443   121.056   180.000  0.154600
  18  O4    os    E   17  15  14     1.363   119.649    -0.000 -0.139200
  19  C5    cd    M   17  15  14     1.382   130.312   180.000 -0.263300
  20  H5    ha    E   19  17  15     1.082   126.031     0.000  0.203100
  21  C6    cd    M   19  17  15     1.417   106.671   180.000 -0.168700
  22  H6    ha    E   21  19  17     1.079   128.927   180.000  0.194700
  23  C7    cc    M   21  19  17     1.368   105.038    -0.000  0.135400
  24  N3    no    M   23  21  19     1.429   130.147   180.000  0.703400
  25  O3    o     E   24  23  21     1.228   118.186   180.000 -0.430100
  26  O2    o     M   24  23  21     1.236   115.541     0.000 -0.430100


LOOP
   N1   C8
   C7   O4

IMPROPER
   N1   N4   C8   O5
   C8   C1   N4   H1
   C2   N4   C1   O1
   C4   H4   C3   N2
   C5   C3   C4   O4
   C4   C6   C5   H5
   C7   C5   C6   H6
   C6   N3   C7   O4
   C7   O3   N3   O2

DONE
STOP
```

frcmod.NFT

```
remark goes here
MASS

BOND

ANGLE
```

```
DIHE
n -n2-ce-h4   1    4.150       180.000           2.000      same as X -c2-n2-X
n -n2-ce-cc   1    4.150       180.000           2.000      same as X -c2-n2-X
n2-ce-cc-os   1    1.000       180.000           2.000      same as X -ce-ce-X
n2-ce-cc-cd   1    1.000       180.000           2.000      same as X -ce-ce-X
ce-cc-os-cc   1    1.050       180.000           2.000      same as X -c2-os-X
h4-ce-cc-os   1    1.000       180.000           2.000      same as X -ce-ce-X
h4-ce-cc-cd   1    1.000       180.000           2.000      same as X -ce-ce-X
cc-os-cc-cd   1    1.050       180.000           2.000      same as X -c2-os-X
cc-os-cc-no   1    1.050       180.000           2.000      same as X -c2-os-X
os-cc-no-o    1    0.750       180.000           2.000      same as X -c2-no-X
cd-cc-no-o    1    0.750       180.000           2.000      same as X -c2-no-X

IMPROPER
n -n -c -o         10.5          180.0         2.0          General improper torsional angle
(2 general atom types)
c -c -n -hn         1.1          180.0         2.0          General improper torsional angle
(2 general atom types)
c3-n -c -o         10.5          180.0         2.0          General improper torsional angle
(2 general atom types)
cc-h4-ce-n2         1.1          180.0         2.0          Using default value
cd-ce-cc-os         1.1          180.0         2.0          Using default value
cc-cd-cd-ha         1.1          180.0         2.0          Using default value
cd-no-cc-os         1.1          180.0         2.0          Using default value
cc-o -no-o          1.1          180.0         2.0          Using default value

NONBON
```
